# Spasm of the Glottis

**Published:** 1909-06-05

**Authors:** 


### Laryngology and Rhinology.

### SPASM OF THE GLOTTIS.

The effect of spasmodic contraction of the laryngeal muscles is to produce adduction of the cords; for the abductor muscles, which probably participate in the contraction, are overcome by the more powerful adductors. In addition to the adduction of the cords there is closure of the ventricular bands, and probably also, in any strong spasm, contracture of the upper aperture of the larynx. Experimentally, stimulation of the centripetal fibres of the vagus of one side produces a reflex adduction of both vocal cords, and stimulation of the peripheral end of the divided recurrent laryngeal nerve causes contraction of the muscles of the same side; but clinically, spasm of the glottis is always bilateral.

The causes of spasm may be distinguished as peripheral and central; and the latter may be divided into organic and functional. Peripheral causes may be situated in or near the larynx and include tumours, foreign bodies, and inflammations of the larynx or adjacent parts; more rarely the spasm is due to irritation of the nerves by an aneurysm or other growth in the neck or thorax. Central organic causes include the laryngeal crises of tabes and general paralysis. Spasm, the result of functional disturbance, is not very uncommon, is associated with hysteria and neurasthenia, and is often excited by some sexual trouble.

A typical severe spasm begins with a sensation of tickling in the throat, followed by a fit of coughing; the glottis then closes tightly and the violent attempts at inspiration are ineffectual. There is generally loud stridor; but when the glottis is completely closed there is no sound, and these are, of course, the worst cases. There is a terrible feeling of oppression and anxiety, and the patient clutches at himself or surrounding objects. As the spasm passes off the air enters with a loud crowing noise and the breathing becomes normal. There may be considerable cyanosis, and in the worst cases there is temporary loss of consciousness; but a fatal result is extremely rare unless some organic cause of obstruction is present. These attacks may recur at variable intervals for years, but tend to improve, and to cease eventually. Often the spasm is less complete, but lasts longer, so that stridor may persist for several hours.

Children are much more liable than adults to spasm of the glottis, which in them often accompanies an ordinary catarrhal laryngitis, and is then known as "spasmodic laryngitis" or "laryngitis stridula." The appearance is that of an ordinary catarrh with a cough, hoarseness, and slight fever. The attack of spasm nearly always comes on in the middle of the night, and usually recurs for the next few nights with diminishing severity. In laryngeal diphtheria the symptoms, which are also more marked at night, tend to get worse, and there is no complete freedom from dyspnœa between the attacks. In spasmodic laryngitis, again, hoarseness and catarrhal symptoms remain between the spasms, and serve to distinguish the affection from "laryngismus stridulus." This is simply spasm of the glottis, which occurs in unhealthy, ill-nourished children, usually between the ages of six months and two years, and generally in association with rickets. It is due to an increased excitability of the nervous system, and the spasm is excited reflexly by some disturbance within the alimentary canal, such as worms or gastritis, or from the pharynx, especially from the presence of adenoids. The attack is exactly similar to the spasm already described; it begins with a few short noisy inspirations, and ends with a long crowing. It is often accompanied by carpo-pedal contractions, and the "silent" cases are the most severe. The attacks vary much in frequency, and occasionally have a fatal termination. In common with all stridulous affections it has been included under the title of "false croup," and nurses speak of it as a "passion-fit" or "holding the breath." The account of the attack may suggest epilepsy if the patient is not seen during the spasm; but in the latter respiration is not completely interrupted, there is no crowing, the mouth is clenched, and there are usually general convulsions.

Two other rare varieties of spasm may be mentioned. "Phonic spasm," or "dysphonia spastica," is an occupation-neurosis allied to writer's cramp, which affects nervous subjects, especially those engaged in professional use of the voice. On attempted phonation the cords come tightly into contact, so that no air passes out and no sound is produced. The spasm generally subsides as soon as the subject ceases to try to speak, but sometimes it persists and induces an ordinary attack of glottic spasm. In less severe cases sounds can be produced intermittently, and the vowels are often doubled, thus superficially resembling stammering.

The second variety is often incorrectly termed "laryngeal vertigo," or, more properly, "ictus laryngis." The attack begins with a cough, is followed by glottic spasm, and then by loss of consciousness, which is regained in a few seconds. There is a close resemblance to *petit mal*, but the onset is always with cough and glottic spasm; there is no after-confusion or stupor, and it occurs in people who never have any other form of fit. The affection is probably merely a variety of glottic spasm, and the loss of consciousness due to the closure of the glottis, for it is well known that forcible expiration against resistance will produce a similar effect.

Among spasmodic affections we may place the nervous cough or "barking cough of puberty"; it is of the nature of a "*tic convulsif*," and affects young people of both sexes. The cough is loud, single, without expectoration, and recurs persistently, ceasing during sleep or if the attention is distracted. Before making the diagnosis, any cause of irritation must be excluded, for a very similar cough may occur in patients of all ages as a result of reflex irritation. The source of irritation which causes such a reflex cough may be situated in any part of the respiratory tract, in the ear, the alimentary canal, or in the bladder. The well-known "stomach cough" is a familiar instance.

